# Excitonic, Optical, and Photovoltaic Properties of the 1T-NiO_2_ Monolayer

**DOI:** 10.1021/acsomega.5c12803

**Published:** 2026-03-27

**Authors:** Israel da Silva Oliveira, Bill D. Aparicio-Huacarpuma, Carlos M. de Oliveira Bastos, Mariana Lumi Ichihara Sado, Alysson M. Almeida Silva, Luiz Antônio Ribeiro Júnior, Alexandre Cavalheiro Dias

**Affiliations:** † Institute of Physics, 564113 University of Brasília, Brasília 70919-970, DF, Brazil; ‡ Computational Materials Laboratory, LCCMat, Institute of Physics, 28127 University of Brasília, Brasília 70910-900, Brazil; ¶ Institute of Physics and International Center of Physics, 564113 University of Brasília, Brasília 70919-970, DF, Brazil; § Department of Mechanical Engineering, University of Brasília, College of Technology, Brasília 70910-900, Federal District, Brazil; ∥ Institute of Physics, 28127 University of Brasília, Brasília 70910-900, Federal District, Brazil; ⊥ Computational Materials Laboratory, LCCMat, Institute of Physics, 28127 University of Brasília, Brasília 70910-900, Federal District, Brazil

## Abstract

Developing new two-dimensional materials for photovoltaics is a central strategy to address the world’s growing energy demands. Herein, we made a multilevel, first-principles computational investigation focused on the characterization of the 1T NiO_2_ monolayer, evaluating its structural stability, vibrational modes and Raman spectrum, electronic, mechanical, and optical properties. Our investigation was done through first-principle calculations based on density functional theory for structural and ground state properties, complemented by many-body perturbation theory to accurately capture quasiparticle (*G*_0_ *W*_0_) and excitonic effects, the latter being calculated with a maximally localized Wannier function-based tight-binding framework to describe the single particle states to solve the Bethe–Salpeter equation. Our calculations confirm that the 1T-NiO_2_ monolayer is energetically, dynamically, thermally (at 300 K), and mechanically stable. We found an indirect electronic band gap of 2.20 eV at the *G*_0_ *W*_0_ level. Furthermore, the optical properties are dominated by strong electron–hole interactions, resulting in a direct excitonic state at 1.34 eV and an exceptionally high exciton binding energy of 880 meV. Although this optical gap is ideally positioned for solar absorption, leading to a theoretical power conversion efficiency (PCE^SQ^) limit of 32.66%, the high exciton binding energy makes exciton dissociation into free charge carriers unfavorable. Despite the strong light absorption, the highly excitonic nature of the 1T-NiO_2_ monolayer makes it unsuitable for conventional photovoltaic applications but potentially promising for exciton-based optoelectronics or photocatalytic devices.

## Graphical abstract

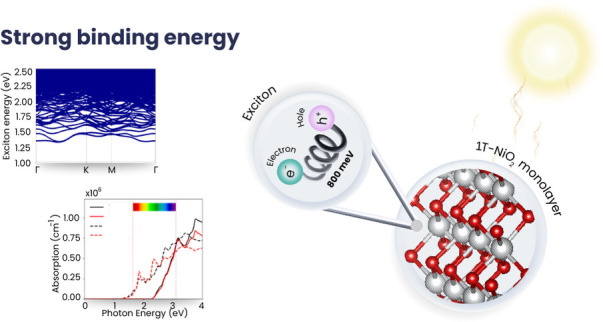

## 1 Introduction

Driven by a growing population and global economic expansion, the energy demand continues to steadily increase.^1^ Although technological advancements and new legislation have promoted gradual improvements in energy production efficiency, these gains often prove inadequate to offset the magnitude of growing energy demand.^2^ This context makes the development of new, sustainable materials for energy production a strategic priority. The experimental isolation of graphene in 2004 ^3^ opened the path for the production of several other two-dimensional (2D) materials with potential applications in the field of optoelectronic and solar harvesting devices.^4–6^ More specifically, in the field of energy generation, the potential of 2D materials lies in the possibility of thinner, lighter, and more flexible solar cells, which can increase production volume and lead to roll-to-roll processing.^7^

Efforts must be made to characterize the structural and energetic properties^8^ of materials in order to assemble a catalog so that, when considering the design of solar cells (or other devices such as batteries,^9^ sensors,^10^ among others), the most interesting materials are readily available in terms of efficiency,^11^ production, and life cycle impact.^12^ From this perspective, the viability of materials for a sustainable future depends not only on their origin (whether they are ‘green’ or renewable) but also on a complex balance among their relative abundance, processing efficiency, and, critically, their final performance.^13^ The introduction of 2D materials is critical in this regard: they enable the use of reduced quantities of material to achieve remarkable electronic and optical properties.^14^ Thus, elements such as transition metals, although nonrenewable, become viable candidates if (a) their 2D form allows for the fabrication of radically more efficient devices^15^ and (b) the material footprint per unit of energy generated is drastically reduced.^16^

Several classes of two-dimensional materials have been previously explored, including MXenes,^17,18^ transition-metal dihalides (TMDHs),^19,20^ Janus structures,^21–23^ and others.^24–27^ Also noteworthy are the transition-metal dichalcogenides (TMDCs),^5,28,29^ which have been extensively investigated in the past decade, where the excitonic effects play a major role in an accurate description of their linear optical response, making them useful for several applications in nanoelectronic,^30^ solar harvesting,^5,31^ and spintronic devices.^32^ Methodologies reported using density functional theory (DFT) for electronic and optical properties using PBE, HSE06, *G*_0_ *W*_0_, and BSE methods for 2D systems are crucial for studying these properties and applications.^33,34^

In this scenario of seeking high-efficiency new 2D materials for solar harvesting applications, this work characterizes the 1T nickel dioxide (NiO_2_) monolayer, particularly with regard to its optical properties and power conversion efficiency (PCE). The aforementioned material is part of the transition-metal dioxide (TMDO) family,^35^ characterized by its lamellar structure and MO_2_ stoichiometry, where M is a transition metal. Other members of this family include OsO_2_,^36^ RuO_2_,^37^ among others. The composition of each individual layer is established by O-M-O bonds, with a layer of oxygen atoms sandwiching the central plane of transition metal atoms.^38^

Herein, we employ a multilevel framework focused on the theoretical description of 1T-NiO_2_ monolayer structural and optoelectronic properties. The ground state was described by DFT, and the description of the electronic levels is improved by many-body perturbation theory (MBPT) via the *G*_0_ *W*_0_ approximation and the Bethe-Salpeter Equation (BSE), thereby providing an accurate description of the band structure and excitonic effects. Furthermore, we evaluate the dynamic and thermal stability of the monolayer and estimate its spectroscopic limited maximum efficiency (SLME) to assess its viability for next-generation solar cells.

## 2 Theoretical Framework and Computational Methods

The structural and optoelectronic properties of the 1T-NiO_2_ monolayer were investigated using first-principles simulations based on the DFT^39,40^ framework. The projector-augmented wave (PAW)^41,42^ formalism was used to calculate the ground-state properties, through *Vienna Ab initio Simulation Package* (VASP),^43,44^ using the Perdew–Burke–Ernzerhof (PBE)^45^ exchange-correlation functional. The plane-wave basis set cutoff energy was adjusted according to the required precision for each property. For geometry optimization, we used a cutoff energy of 933 eV, for elastic constants, in order to achieve a good precision, we increased to 1167 eV, for AIMD, due to the high computational cost, we used 480 eV, and for other properties, we used 525 eV.

A Γ-centered Monkhorst-Pack scheme^46^ was used for the Brillouin zone integrations, with a **k**-mesh of 16 × 16 × 1 for structural relaxation and other properties, except for the density of states (DOS) calculation, a denser grid of 32 × 32 × 1 was employed. A total energy criterion of 1 × 10^–6^ eV was applied for the Kohn–Sham (KS) self-consistent cycle. The equilibrium structures were obtained by stress tensor optimization and minimization of interatomic forces until the atomic forces on each atom were less than 0.01 eV Å^–1^. Moreover, we used a vacuum of 16.89 Å in the nonperiodic direction to avoid spurious interactions.

We used *ab initio* molecular dynamics (AIMD) simulations to evaluate the thermodynamic stability of a 1T NiO_2_ monolayer, using the FHI-aims code,^47,48^ which employs a numerical atom-centered orbitals (NAOs) basis set. Our simulations used a light first-tier NAOs basis set, at NVT ensemble with Nosé-Hoover thermostat, at 300 K during 10 ps with a time step of 1 fs, with a 3 × 3 × 1 supercell using the 4 × 4 × 1 **k**-points density.

To accurately capture the quasiparticle and excitonic effects critical for optical properties, we employed MBPT.^49^ The electronic band gap was first corrected at the *G*_0_ *W*_0_ ^50,51^ level of approximation, addressing the known underestimation by the PBE functional.^52,53^ These calculations were initialized from a PBE ground state and included 128 electronic bands, with a 48 × 48 × 1 **k**-points mesh and a plane-wave basis cutoff of 525 eV.

For the optical and excitonic properties, the Maximum Localized Wannier Functions (MLWFs) are generated considering p orbital projections for O and d orbital projections for Ni, complemented by a random basis set to achieve the 128 bands used in *G*_0_ *W*_0_ corrections DFT calculations and to have a better treatment with Ni-O hybridized states through the Wannier90 package.^54^ The BSE^55^ was solved using the WanTiBEXOS code,^56^ being the electron–hole single particle states described by an MLWF parametrized Tight-Binding (TB) Hamiltonian, straight obtained from the Wannier90 code.^54^ These BSE simulations used a **k**-points density of 120 Å^–1^, considering the four highest valence bands and the two lowest conduction bands, which are enough to describe the linear optical response in the solar emission range (i.e., 0–4 eV).^57^ The electron–hole Coulomb interaction was modeled using a two-dimensional truncated Coulomb potential (V2DT) to account for dimensional confinement.^58^ A Gaussian smearing σ = 0.05 eV was applied to obtain the real and imaginary parts of the dielectric function. This MLWF-TB+BSE used in this work to simulate excitonic and optical properties reduces the computational cost significantly, when compared to the pure *ab initio* method to solve BSE. Another important point is that the calculation of the exciton band structure, used to identify possible indirect excitonic ground state, is only implemented in the WanTiBEXOS package^56^ that used MLWF-TB+BSE method.

Finally, the potential of 1T-NiO_2_ for photovoltaic applications was evaluated by estimating its PCE at the Shockley-Queisser (SQ) limit^59^ and SLME model,^60^ with WanTiBEXOS code.^56^ While the SQ limit relies solely on the fundamental gap, the SLME model also incorporates the material’s absorption spectrum and fundamental and direct band gap. In these calculations, the solar emission spectrum was modeled by AM1.5G solar irradiance spectrum^57^ at an operating temperature of 300 K. The absorbance rate was obtained by the total absorption coefficient, obtained by the diagonal parts of the dielectric tensor and considering an effective monolayer thickness defined as its intrinsic value plus a van der Waals (vdW) correction of 3.3 Å, following the methodology proposed by Bernardi et al.,^16^ which previously shows good agreement with experimental data.

## 3 Results and Discussion

### 3.1 Structural Properties and Stability

The 1T-NiO_2_ monolayer crystal structure, shown in Figure 1, assumes trigonal symmetry, with a relaxed lattice parameter of *a*_0_ = 2.82 Å, a Ni–O distance of 1.88 Å, and a monolayer thickness of 1.89 Å. The lattice angles take the following values: α = β = 90° and γ = 120°. The obtained structural parameters are in good agreement with previous works.^61–63^

**Figure 1:**
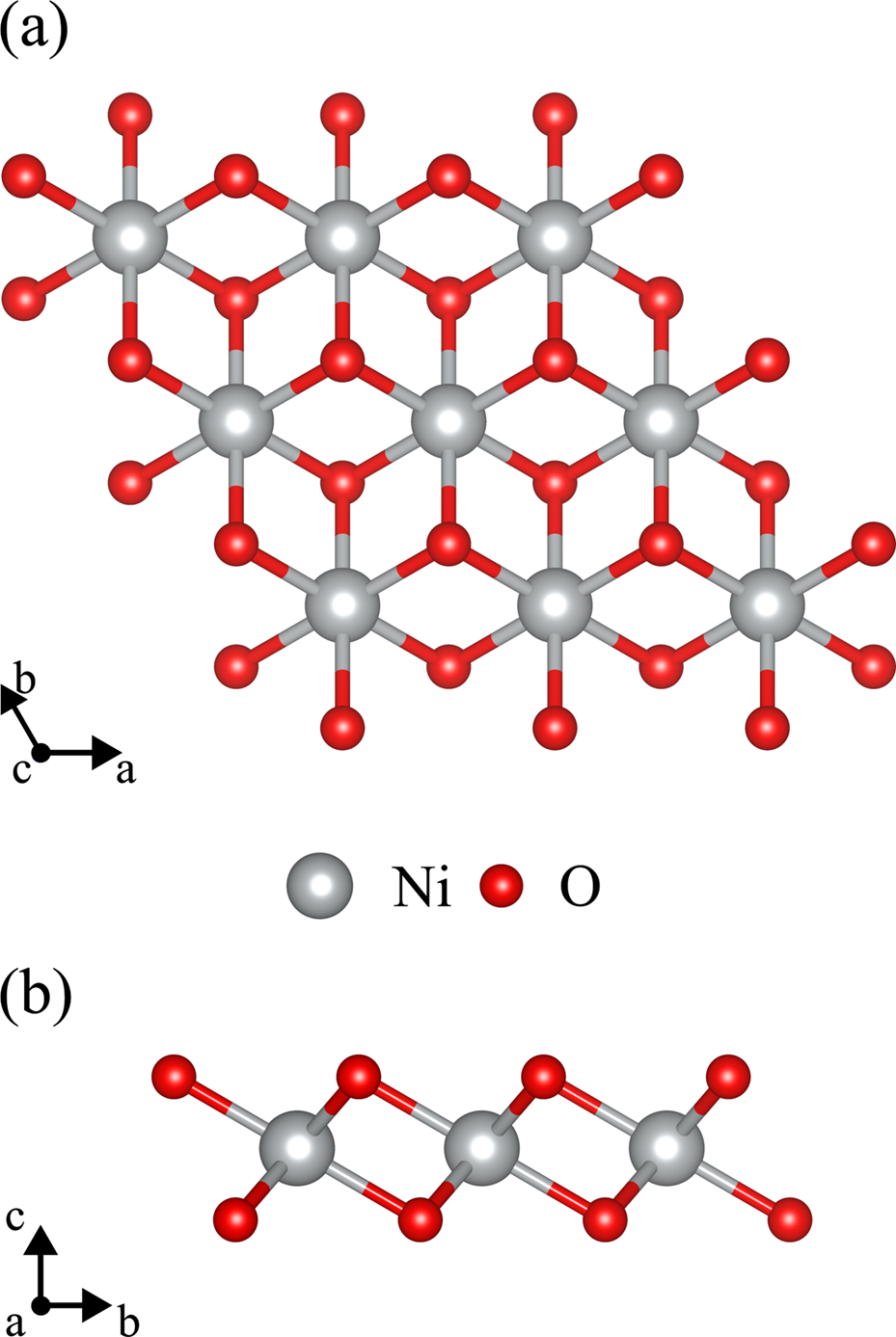
Top (a) and side (b) views of the crystal structure of trigonal 2D-NiO_2_ monolayers with a 3 × 3 × 1 supercell.

The 1T phase originates from a structural reconfiguration of the 2H phase, characterized by lateral sliding of one of the atomic planes of anions (X). In this geometry, the top projection reveals that the displaced atoms now occupy the centers of the hexagonal rings defined by the adjacent M-X subnetwork.^64^ In the context of NiO_2_, the 1T phase (space group $P\overset{-}{3}m1$ ) represents the structural configuration, characterized by the octahedral coordination of nickel atoms encapsulated between oxygen layers. In this arrangement, the NiO_6_ octahedra share edges, forming a triangular network.

Regarding the monolayer stability, we analyzed four aspects: energetic, dynamical, thermal, and mechanical stability. In a later section, the phonon dispersion is discussed in the context of vibrational properties. First, the energetic stability of the crystal was evaluated based on its cohesive energy (*E*_coh_), which represents the energy required to separate the solid into its neutral and isolated constituent atoms. The calculation was performed using the following expression:

$$E_{\mathrm{coh}}=\frac{E_{\mathrm{tot}}-\sum_{i}N_{i}E_{i}}{N_{\mathrm{atoms}}}$$ 1

where *E*_tot_ represents the total energy of the monolayer, *N*_*i*_ is the number of atoms of species *i* (Ni or O), *E*_*i*_ is the total energy of the isolated atom of type *i*, and *N*_atoms_ is the total number of atoms in the unit cell. The calculated cohesive energy for the 1T-NiO_2_ monolayer was −4.38 eV per atom. A negative value for *E*_coh_ confirms that the structure is energetically stable with respect to its isolated constituent atoms, indicating the presence of strong interatomic bonds.

The thermodynamical stability of 1T NiO_2_ was established through AIMD simulations run at room temperature (300 K), using the canonical NVT ensemble, for 10 ps. The total energy fluctuations during the simulation time are depicted in Figure 2. Following a brief transient period (≲1 ps), the total energy remains in the vicinity of a steady mean value throughout the 10 ps trajectory, exhibiting no systematic drift. From 1 to 10 ps, the total energy small oscillations indicate the absence of bond breaking or reconstruction events.

**Figure 2:**
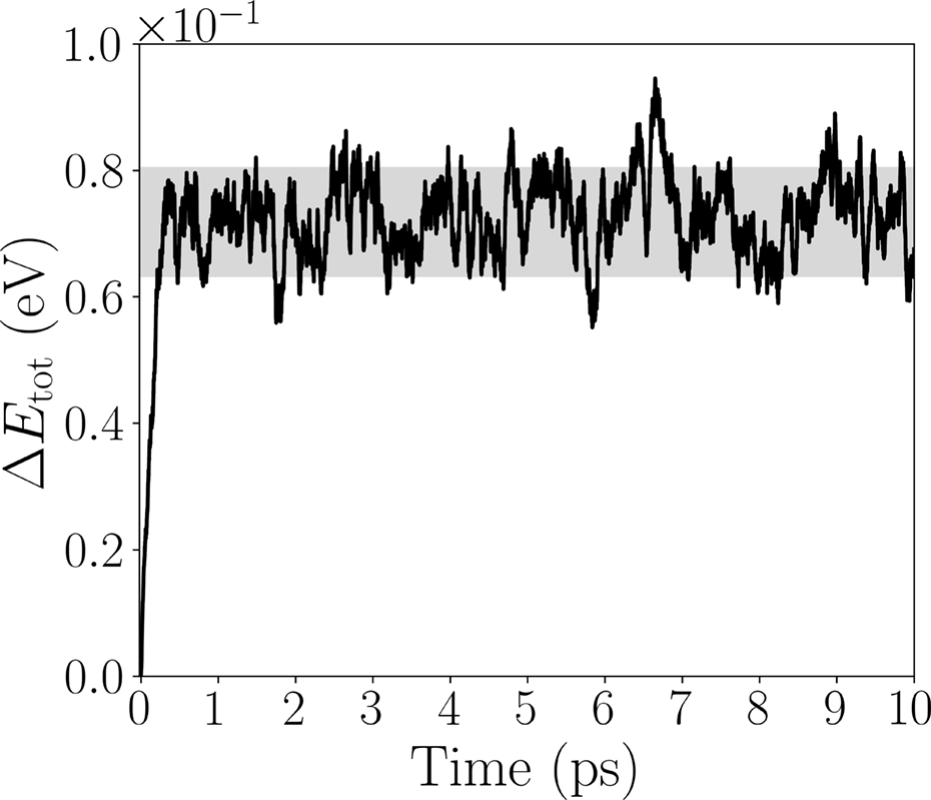
Time-dependent AIMD simulation for the NiO_2_ monolayer at 300 K and 10 ps.

The NiO_2_ monolayer mechanical stability was assessed through Born’s stability criterion,^65,66^ which requires *C*_11_ > 0 and *C*_11_^2^ > *C*_12_^2^, being satisfied in this monolayer, confirming its mechanical stability. Owing to the hexagonal symmetry constraints, only two independent elastic constants are required: *C*_11_ = *C*_22_ and *C*_12_ = *C*_21_.

1T-NiO_2_ monolayer is intrinsically stable and technologically promising, and there are multiple experimentally proven routes. The most feasible route is chemical vapor deposition (CVD), which is generally used on glass, sapphire or quartz substrates. It was previously used to obtain MoS_2_.^67^ Also exfoliated methods can be an alternative to obtain with 1T-NiO_2_ controlled airflow or pressures, as suggested for 1T-V_2_O_3_ and 1T-WS_2_ films.^68^ Previously, NiSe_2_ were synthesized direct selenylation of the nickel substrate after epitaxial growth of selenium on a nickel foil via external thermal annealing at around 473–523 K.^69^ This background leads us to believe that the proposed material can be successfully synthesized through reverse engineering and combining deposition methods.

After reviewing and synthesizing a possible 2D NiO_2_ system, it is worth noting that a phase transition from NiO_2_ to Ni­(OH)_2_ may occur in the presence of moisture.^70^ Therefore, protective layers are recommended to prevent the formation of hydroxide phases, which would alter the expected cleavage energies and structural and electronic properties.

Also, due to the layered form of NiO_2_, in materials such as TMDs, layer-by-layer (turbostatic) stacking is used to induce or increase the degree of noncentrosymmetry,^71^ resulting in lattice mismatches that naturally lead to a disordered and rotated structure. Similar to graphene,^72^ this arrangement promotes electronic decoupling between layers, allowing the intrinsic properties of the 1T-NiO_2_ monolayer to be investigated without the interference of orbital hybridizations.

### 3.2 Mechanical Properties

As previously mentioned in the structural stability analysis, there are only two independent in-plane elastic constants due to the monolayer hexagonal symmetry. These are *C*_11_ = *C*_22_ and *C*_12_ = *C*_21_, while the shear constant is given by *C*_66_ = (*C*_11_ – *C*_12_)/2. The mechanical properties were investigated by the Young’s modulus (*Y* (θ)) and the Poisson’s ratio (ν­(θ)). The mathematical formulation for these quantities follows as:

$$Y(\theta )=\frac{C_{11}^{2}-C_{12}^{2}}{C_{11}(\sin^{4}\theta +\cos^{4}\theta )+\left(\frac{C_{11}^{2}-2C_{12}}{C_{66}}\right)\cos^{2}\theta \sin^{2}\theta }$$ 2

$$\nu (\theta )=\frac{C_{12}^{2}-\left(C_{11}+C_{12}-\frac{C_{11}^{2}-C_{12}^{2}}{C_{66}}\right)\cos^{2}\theta \sin^{2}\theta }{C_{11}(\sin^{4}\theta +\cos^{4}\theta )+\left(\frac{C_{11}^{2}-2C_{12}}{C_{66}}\right)\cos^{2}\theta \sin^{2}\theta }$$ 3

The average value of the Young’s modulus obtained was 138.15 Nm^–^^1^, with a standard deviation on the order of 10^–5^ Nm^–^^1^. For Poisson’s ratio, we obtained an average value of 0.23, with a standard deviation of 10^–7^. As a comparison of Young’s moduli with other 2D nanosystems, we can note that MoS_2_ has ≃200 Nm^–^^1^,^73^ NiP_2_ with ≃125 Nm^–^^1^,^74^ CrO_2_ with ≃236 Nm^–^^1^,^75^ and 1T-SnO_2_ with ≃120 Nm^–^^1^.^76^ It is observed that the 2D 1T-NiO_2_ with ≃138 Nm^–^^1^ is lower than MoS_2_ and CrO_2_, but higher than NiP_2_ and SnO_2_.

The polar plot shown in Figure 3, along with the absolute values for each parameter *Y*(θ) and ν­(θ), demonstrates a high degree of directional isotropy in the monolayer’s elastic response. The polar plots of key mechanical parameters, including Young’s modulus (*Y*(θ)) and Poisson’s ratio showing isotropic (ν­(θ)) shape with the angle, consistent with other 2D materials.^77,78^

**Figure 3:**
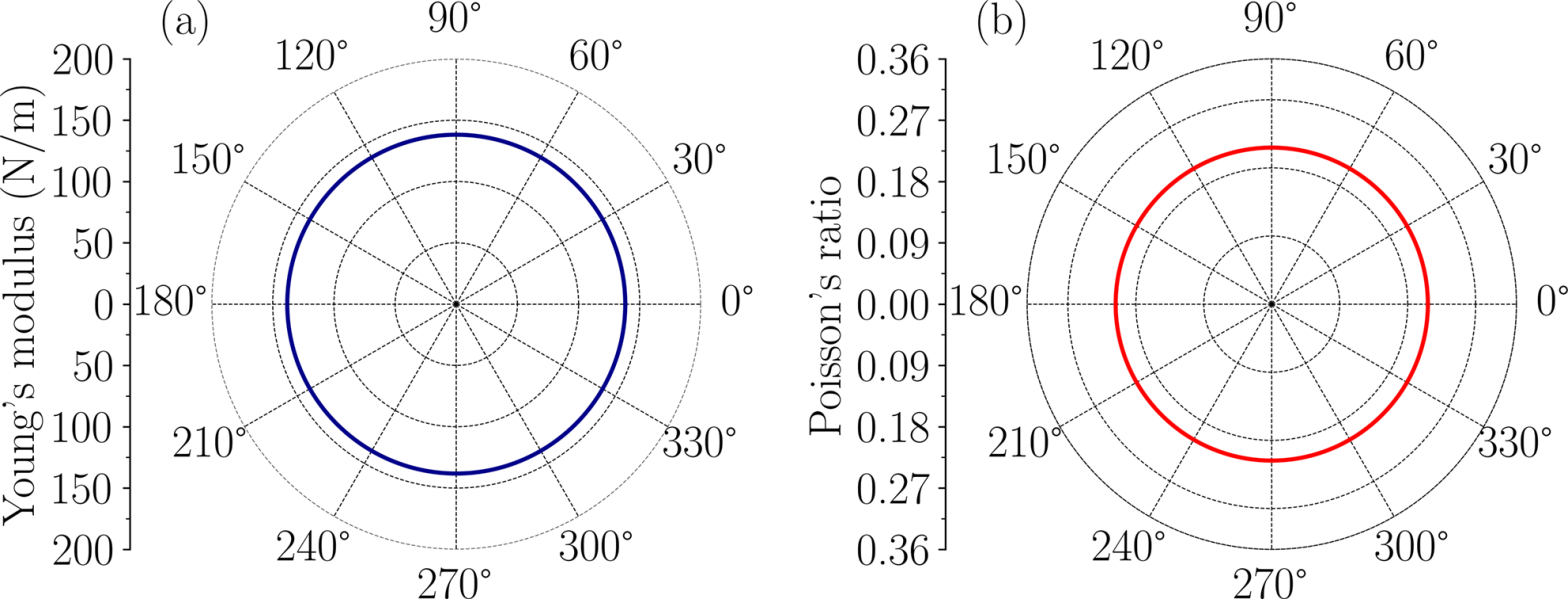
Polar diagrams of (a) Young’s modulus and (b) Poisson’s ratio for the 1T NiO_2_ monolayer, illustrating its isotropic mechanical response.

### 3.3 Phonon Dispersion and Vibrational Properties

The phonon dispersion of the 1T-NiO_2_ monolayer is shown in Figure 4a. As expected, we have 9 vibrational modes, with 3 considered acoustic modes and 6 optical modes, as illustrated in Figure 4a. Considering the crystal structure of the NiO_2_ monolayer consisting of three atoms, the optical modes at the Γ point of the Brillouin zone can be classified as follows: Γ­(NiO_2_) = *E*_g_ + *A*_1g_ + *A*_2u_ + *E*_u_, where the *E*_g_ and *E*_u_ are doubly degenerate modes vibrating in-plane, meanwhile the *A*_1g_ and *A*_2u_ are modes vibrating out-of-plane, consistent with the reference.^79^ As shown in Figure 4b, the NiO_2_ presents modes at 477.46 cm^–1^ (×2), 550.80 cm^–1^ (×2), 556.72 cm^–1^, and 637.68 cm^–1^, respectively. It is found that the *E*_g_ and *A*_1g_ phonon modes are Raman active modes corresponding to optical modes (at 477.46 cm^–1^ (×2) and 556.72 cm^–1^), the Raman inactive modes at 550.80 and 637.68 cm^–1^ have the symmetry *E*_u_ and *A*_2u,_ respectively. These results are confirmed by the Raman spectrum in Figure 4c. The absence of imaginary frequencies in the phonon dispersion also suggests the dynamic stability of this monolayer.

**Figure 4:**
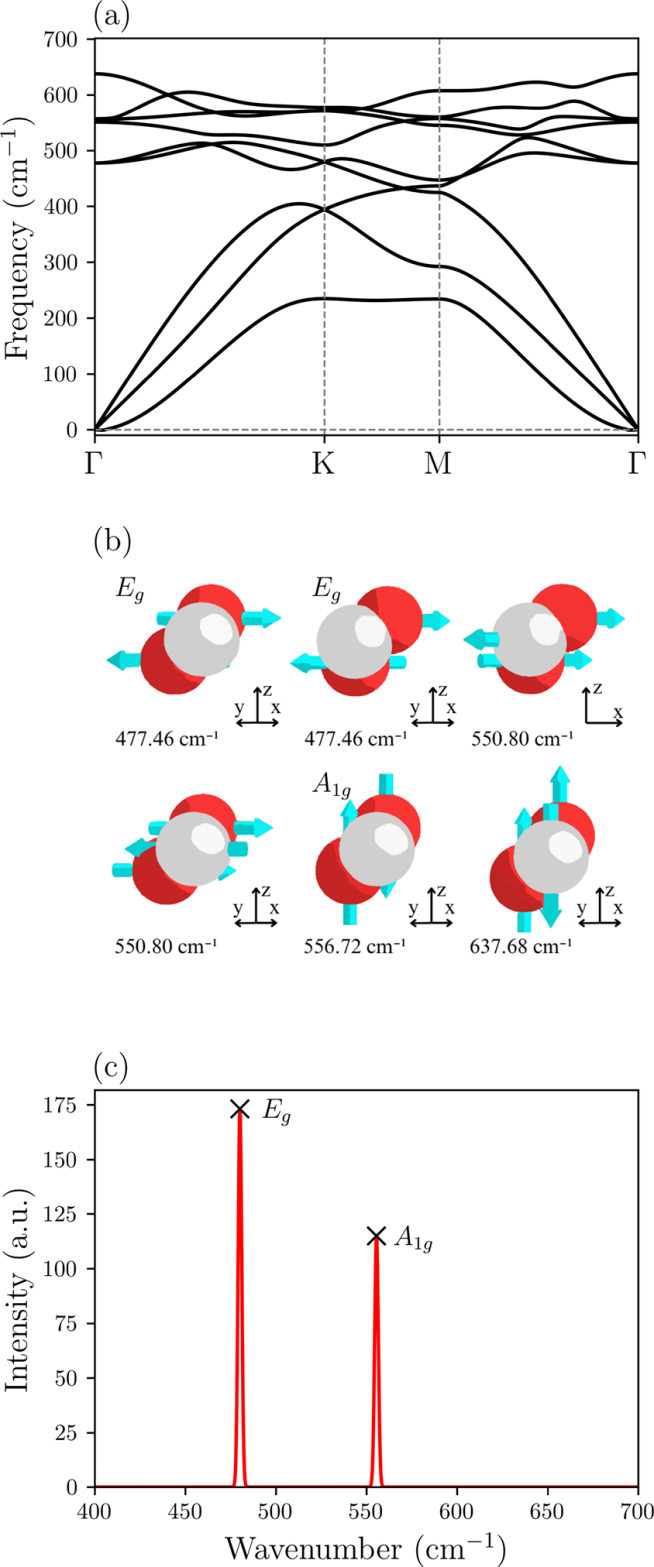
Vibrational properties of the NiO_2_ monolayer, calculated within the PBE functional. (a) Phonon dispersion. (b) Visualization of the Γ-point phonon modes, identifying the Raman-active modes and their respective frequencies. (c) Raman spectrum at the Γ-point.

### 3.4 Electronic Properties

The PBE electronic band structure and density of states of the NiO_2_ monolayer are shown in Figure 5a,b, respectively. A comparison between the PBE and *G*_0_ *W*_0_ band structure are shown in Figure 5c. We have an indirect PBE­(*G*_0_ *W*_0_) fundamental band gap of 1.38 eV­(2.20 eV). In both cases, the conduction band minimum (CBM) is located along the M – Γ path, and the same holds for the valence band maximum (VBM), which is also found along the M – Γ path. We also obtained a PBE­(*G*_0_ *W*_0_) direct band gap, between M – Γ, of 1.59 eV­(2.41 eV). The *G*_0_ *W*_0_ correction is based on the MBPT, which corrects each eigenvalue obtained from the PBE calculation, resulting in a band gap closer to experimental values. The obtained band structures are consistent with previous theoretical studies.^61–63^

**Figure 5:**
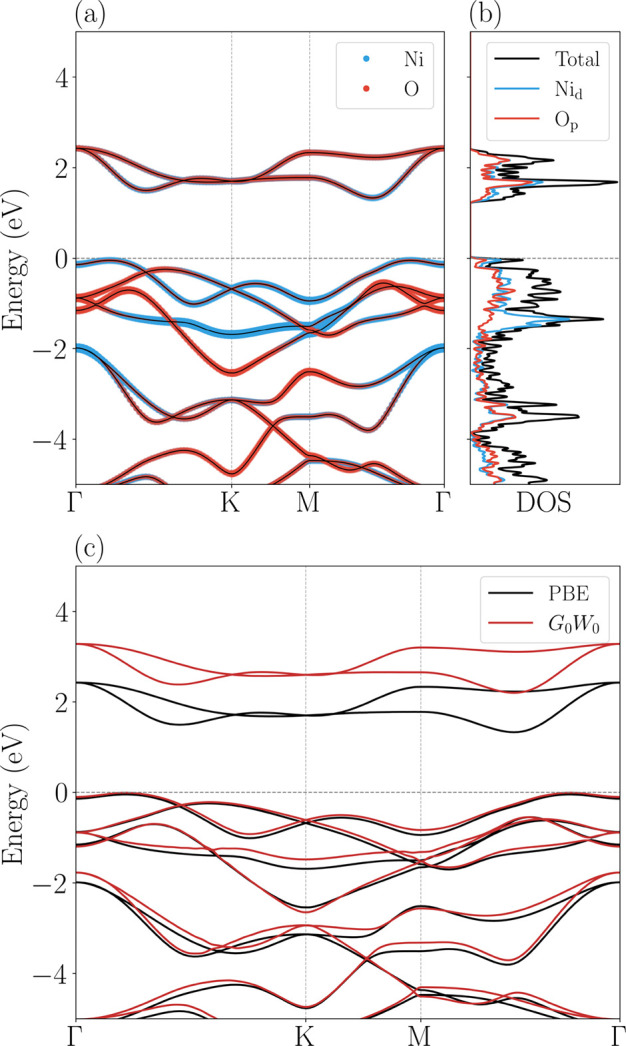
(a) Projected electronic band structure showing the contributions of Ni and O atoms, calculated within the PBE functional. (b) Total and projected density of states (DOS), displaying the dominant orbital contributions of Ni and O at the PBE level. (c) Comparison of PBE (black curves) and *G*_0_ *W*_0_ (red curves) electronic band structures.

The analysis of the projected electronic bands and density of states (PDOS), shown in Figure 5a,b, respectively, allows the identification of specific contributions from atoms to each band, and the PDOS supports the analysis with orbital contributions. It is observed that the valence bands closest to the Fermi level are predominantly formed by Nickel d-orbitals (Ni_d_), indicated in blue in Figure 5b. There is, however, significant hybridization with Oxygen p-orbitals (O_p_), represented in red, suggesting a strong covalent interaction between Nickel and Oxygen atoms. On the other hand, the lowest conduction bands are strongly hybridized, with visible contributions from both atoms. The observed p–d hybridization is quite expected in undoped films,^80^ but contrasts with NiO, where the valence band is dominated by O 2p states and the conduction band by Ni 3d states.^80,81^ This mixing, for these low-energy unoccupied states, governs the material’s electronic and optical properties.

Therefore, in the 1T-NiO_2_ structure, each Nickel atom is subject to an octahedral crystal field generated by the surrounding Oxygen atoms. Since Ni is in a formal + 4 oxidation state, the octahedral field splits the Ni-3*d* orbitals into triply degenerate *t*_2g_ levels (lower energy) and doubly degenerate *e*_g_ levels (higher energy). As described by the DOS, the valence band edge is dominated by occupied *t*_2g_ states hybridized with the O-2*p* orbitals, while the conduction band is formed mainly by *e*_g_ states. As a result, the band gap is a consequence of this crystal field splitting stabilized by the low-spin configuration of the *d* ^6^ cations. As the fundamental electronic gap is indirect, we suggest that strong *p*-*d* hybridization identified in the DOS has a crucial role in the dispersion of these bands, governing the material’s electronic behavior.

### 3.5 Excitonic and Optical Properties

From the exciton band structure, shown in Figure 6, it is noted that the monolayer exhibits an indirect excitonic ground state located along the Γ – K path, with an energy of $E_{x_{\mathrm{gs}}}=1.32\mathrm{eV}$ , which was expected due to the indirect nature of the fundamental electronic band gap; this also allows phonon-assisted optical transition with photon excitation energies lower than the optical band gap obtained from the direct optical transitions. The excitonic binding energy, defined as the difference between the fundamental band gap energy (*E*_g_) and the ground-state excitonic energy $\left(E_{x_{\mathrm{gs}}}\right)$ , was calculated as 0.88 eV, which is higher than the typical values for 2D monolayers.^5,6,82–84^

**Figure 6:**
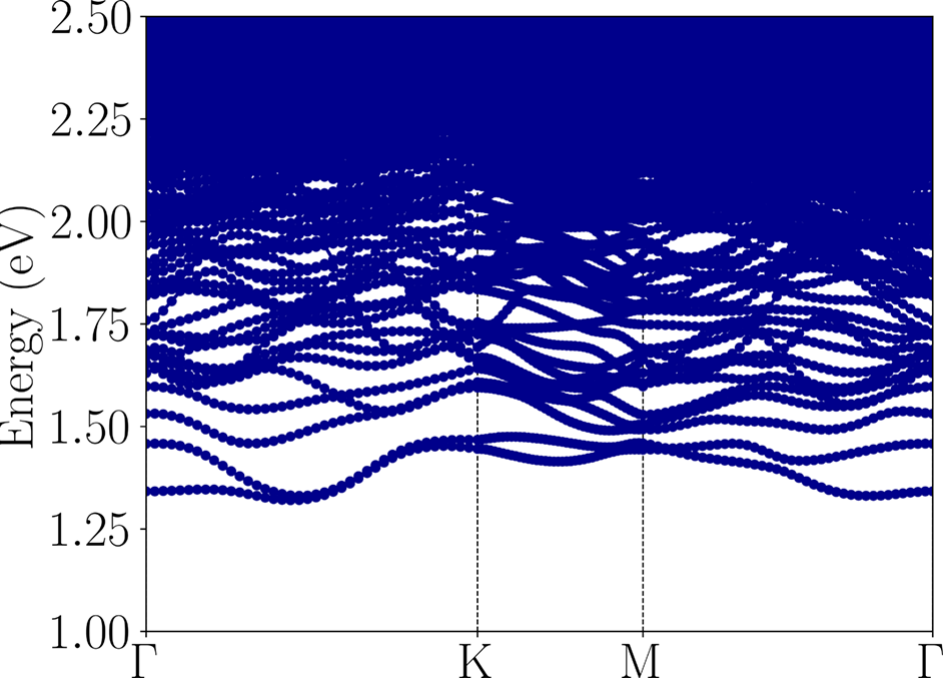
Exciton band structure of the NiO_2_ monolayer obtained from a tight-binding Hamiltonian based on G_0_W_0_. The system exhibits an indirect excitonic ground state with an energy of $E_{x_{\mathrm{gs}}}=1.32\mathrm{eV}$ .

It is also important to understand that excitonic effects, different from the electronic levels, are very sensitive to the surrounding dielectric environment and substrate proximity effects,^5^ which can lower the exciton binding energies, weakening these quasi-particle effects. In this scenario, the BSE results assume a monolayer in a vacuum, which serves as an upper bound for excitonic effects. The exciton binding energy can also be tuned by substrate engineering or heterostructures that could substantially reduce exciton binding and alter the optical band gap. The IPA level is the total absence of these effects; a more realistic linear optical response, from an experimental point of view, lies between IPA and BSE results, as it is common to grow these monolayers on a substrate.

For a better comparison, we can point to other materials such as MoS_2_ and WSe_2_ with values of ≃240 meV experimentally.^85^ Also, theoretically proposed 2D carbon allotropes had exciton energies between 83 and 575 meV.^6^ S-doped graphyne with 460 meV.^86^ In addition, Hf-based MXenes monolayers using *the GW method* were also reported with ≃510 to ≃760 meV^87^ considered high excitonic energies. Also, a direct excitonic state with an energy of $E_{{x_{\mathrm{gs}}}^{d}}=1.34\;\mathrm{eV}\;$ is also observed at the Γ point in Figure 6, this value also represents the optical band gap of this monolayer, as its optical transition is dipole-allowed.

The linear optical response is represented by the absorption coefficient (α), reflectivity (*R*), and refractive index (η) calculated using the IPA (solid lines) and BSE (dashed lines) methods, considering linear light polarization along the *x̂* (black curves) and *ŷ* (red curves) directions, shown in Figure 7. The inclusion of excitonic effects, through the BSE approach, significantly changes the absorption spectrum, Figure 7a, compared with the independent particle approximation (IPA). While IPA shows no absorption in the infrared (IR) region and exhibits quasi-isotropic behavior, the BSE spectrum shows a general increase in absorption in the IR and visible areas. Specifically, the BSE calculation reveals a small optical anisotropy in the visible region, which persists at higher energies. The remarkable absorption intensity throughout this range, slightly higher than expected from band-to-band transitions, is a direct consequence of the electron–hole interaction.

**Figure 7:**
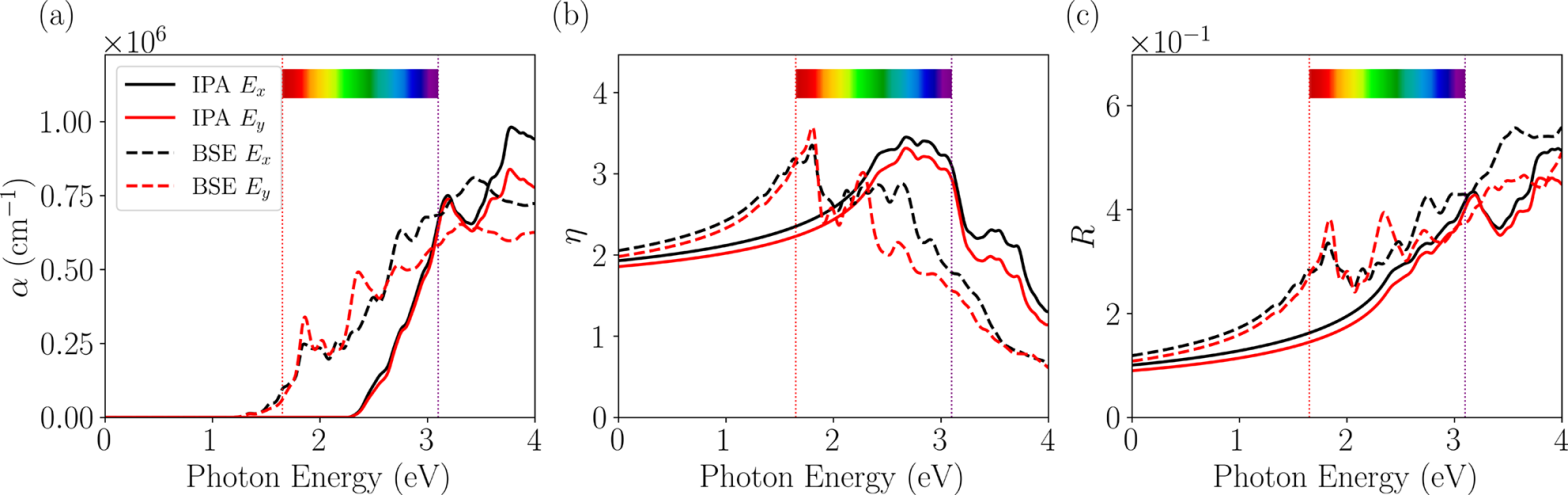
Optical properties of the NiO_2_ monolayer calculated within the IPA (solid lines) and BSE (dashed lines) frameworks: (a) absorption coefficient (α), (b) refractive index (η), and (c) reflectivity (*R*), between 0 and 4 eV of photon energy and for linear light polarization along the *x̂* (black curves) and *ŷ* (red curves) axes.

In the visible spectrum range (1.7–3.1 eV), the material’s refractive index (η), Figure 7b, varies between approximately 2.2 and 3.5 eV, as obtained from the BSE calculations. These values are slightly below those typically reported for other transition metal dichalcogenides, such as MoS_2_, WS_2_, and WSe_2_, whose maximum values exceed 4.0.^88^ A sharp peak near 1.8 eV is observed in the BSE curves, associated with excited excitonic states, while the ground state is located near 1.3 eV. Such a feature is not reproduced in the IPA approximation, which predicts a significant increase in η starting from 2.2 eV. This energy shift of over 0.4 eV indicates strong excitonic binding, influencing the material’s optical response.

The material’s reflectivity (*R*) exhibits significant variation as a function of photon energy and incident-light polarization. According to the results obtained via the BSE calculation (Figure 7c) for the *E*_*x*_ polarization, two main peaks are observed: one at 1.8 eV, with *R* ≈ 0.33, and another at 2.7 eV, with *R* ≈ 0.42. For the *E*_*y*_ polarization, the reflectivity shows maxima at 1.8 eV (*R* ≈ 0.38), 2.3 eV (*R* ≈ 0.40), and 2.7 eV (*R* ≈ 0.36). These spectral features are not reproduced in the IPA calculation, indicating the contribution of excitonic effects, especially in the region near 1.8 eV. In the visible range, the material’s reflectivity varies between approximately 17 and 42% (IPA), being slightly higher at the BSE level, with maximum values comparable to those reported for other transition metal dichalcogenides, such as MoS_2_, whose reflectivity peak reaches about 55%.^89,90^ On the other hand, the obtained values are significantly higher than those observed in 2D Janus materials, such as SnSeS,^22^ whose maximum reflectivity reaches 12% (BSE) and 9% (IPA).

### 3.6 Photovoltaic Performance

The NiO_2_ monolayer exhibited a favorable optical band gap, closer to the maximum efficiency band gap of 1.33 eV for a single photoabsorber at the Shockley–Queisser limit,^59^ despite the very high exciton binding energy of 880 meV, which could lower its solar harvesting efficiency. This indicates that, despite the material’s high optical absorption in the visible range, the conversion of excitons into free charge carriers is highly unfavorable. For example, the thermal energy at room temperature (*k*_B_ *T* ≈ 26 meV) is almost 30 times lower than the exciton binding energy, indicating that the dissociation of excitons into free charge carriers is thermodynamically unfavorable.

In this sense, when estimating the conversion rate of incident light energy into usable electrical power (PCE), we can operate at two commonly adopted levels: the Shockley–Queisser (SQ) limit and the spectroscopy-limited maximum efficiency (SLME). The SQ limit provides the maximum theoretical efficiency^91^ based on a single parameter (the band gap), fundamentally assuming that every absorbed photon above this gap energy generates a free electron–hole pair. The SLME method, in contrast, provides a more detailed limit by incorporating additional material-specific parameters, including the total absorption coefficient, fundamental and direct band gaps (calculated at the IPA level), and exciton states (evaluated at the BSE level).

Considering the high exciton binding energy discussed previously, applying the standard SQ approximation becomes problematic. If one naively uses the direct exciton energy (which governs the primary optical absorption) as the input threshold for the SQ calculation, then a high PCE^SQ^ is obtained, often close to the theoretical maximum. However, this value significantly overestimates the actual efficiency. This discrepancy arises because the calculation relies on the core SQ assumption (100% free-carrier generation), which is fundamentally incorrect for a material where excitons are strongly bound and do not readily dissociate.

That said, the established methodology for the upper limit of the PCE can be summarized in the following formula:

$$\mathrm{PCE}=\frac{J(V_{\max})V_{\max}}{P_{\mathrm{SOLAR}}}$$ 4

where *V*_max_ is the voltage that maximizes the *J*(*V*)*V* product, *J*(*V*) is the current density, described by the following expression:

$$J(V)=J_{SC}-\frac{J_{0}}{fr}(e^{\mathrm{eV}/k_{B}T}-1)$$ 5

where *T* the solar cell temperature, fr is the radiative electron–hole recombination fraction, obtained at IPA level by the difference between direct and fundamental band gap and at BSE level by the difference between direct and fundamental excitonic ground state, *J*_SC_ the short-circuit current density, also known as the photogenerated current, *J*_0_ the reverse saturation current density, defined as:

$$J_{\mathrm{SC}}=e\int_{0}^{\infty }a(E)\frac{P(E)}{E}dE$$ 6

$$J_{0}=e\pi \int_{0}^{\infty }a(E)\Phi_{bb}(E)dE$$ 7

The spectral power distribution *P*(*E*), shown above, is modeled by the AM1.5G solar spectrum, which is the standard reference for terrestrial photovoltaic applications under unconcentrated sunlight.^57^ Φ_bb_(*E*) is the blackbody radiation spectrum, and *a*(*E*) is the absorbance rate, which directly depends on the total absorption coefficient and monolayer effective thickness. More details of the mathematical formalism used for the PCE estimation can be found in the WanTiBEXOS manuscript.^56^

Thus, the calculated PCE values for each approximation level are shown in Table 1. Taking the IPA approximation level calculated using the Shockley-Queisser limit (PCE^SQ^), the photovoltaic efficiency of the monolayer is approximately 14.07%. Now, including exciton effects via the BSE formalism, there is a significant gain in efficiency, reaching 32.66% as predicted, nearly the theoretical limit value.^91^

**Table 1:** Power Conversion Efficiencies for the NiO_2_ Monolayer at *T* = 300 K Compared with Literature Data into Theoretical Models at the Independent Particle Approximation (IPA) and Bethe-Salpeter Equation (BSE) Levels^a^

system	level	PCE^SLME^	PCE_max_^SLME^	PCE^SQ^	ref.
1T-NiO_2_	IPA	0.14	14.56	14.07	this work
	BSE	0.77	31.91	32.66	this work
ScNbCO_2_	IPA		14.33	20.20	^92^
	BSE		20.68	29.27	
ScYC(OH)_2_	IPA	0.45	29.76	29.76	^93^
	BSE	0.32	16.82	23.44	
PdS_2_	IPA	1.03	16.64	18.26	^94^
	BSE	1.28	27.89	28.95	
SiS_2_	IPA		1.56	1.94	^95^
	BSE		2.34	3.02	
SiSe_2_	IPA	0.07	11.52	14.23	^95^
	BSE	0.11	17.63	21.25	
SnSO	IPA	0.52	27.82	27.82	^96^
	BSE	0.45	26.56	30.35	
GeSO	IPA	0.83	31.06	31.06	^96^
	BSE	0.86	28.56	32.46	

a The table contrasts the efficiency limited by the actual spectroscopic absorption (PCE^SLME^) with the ideal limits assuming 100% absorbance (PCE_max_^SLME^) and the Shockley-Queisser limit (PCE^SQ^), both based on the respective energy thresholds (electronic gap for IPA, exciton energy for BSE). All efficiency values are given as a percentage (%).

When we take the SLME model (PCE^SLME^), we see that the predicted efficiency of the monolayer is drastically reduced to less than 1.0% at both approximation levels. Even in this scenario, the contribution of exciton effects is remarkable, increasing the photovoltaic efficiency by more than five times compared to the IPA prediction. The main factor contributing to the material’s low efficiency (PCE), as predicted by the SLME model, is thickness. In the case of monolayers, they are not thick enough to absorb a significant fraction of the incident photons.

To overcome the limitations imposed by 2D materials, we can consider light-harvesting strategies to increase effective absorbance, as proposed by Jariwala and co-workers.^97^ Based on this assumption, we model the absorbance spectrum as a Heaviside step function so that all photons with energy above the optical band range are considered absorbed, obtaining the maximum efficiency of the SLME (PCE_max_^SLME^).

In this case, the NiO_2_ monolayer shows PCE_max_^SLME^ of 14.56% (IPA) and 31.91% (BSE). Both values are very close to their respective SQ limits (14.07 and 32.66%). In fact, the IPA level shows a small gain over the SQ, while the BSE level shows a small loss relative to its limit. There is a brief discussion to be made regarding the gain/loss of efficiency between the PCE_max_^SLME^ and PCE^SQ^ forecasts: at the BSE level, $E_{{x_{\mathrm{gs}}}^{d}}$ was at the optimum point of the SQ limit. When we include the indirect gap and other effects in PCE_max_^SLME^, the effective gap that determines *V*_OC_ is reduced, moving away from the optimum point and causing a loss. For the IPA level, the inverse analysis is valid.

Neither of the theoretical models that evaluate PCE explicitly considers exciton binding energy in their formalisms; as a result, we can expect lower values due to the elevated exciton binding energy of this monolayer. It is also important to understand that the presented PCE values are the upper bound for solar harvesting efficiency, being a huge experimental challenge to achieve these values experimentally, demanding years of research searching for a high-quality synthesis of the monolayer and a good solar cell architecture, seeking other materials to be used as electron transport layer and hole transport layer.

As a comparison of our PCE values using the idealistic SQ method considering electron–hole interaction due to quantum confinement in nanostructured systems, bimetallic ScNbCO_2_ MXene^92^ was recently reported with a PCE-SQ value of 29.76%, considering the excitonic effect. Also, 1T-PdS_2_ was reported with PCE^SQ^ of 28.95% at the BSE level.^94^ Other systems such as SnSO and GeSO were also reported with values of 30.35 and 32.46%,^96^ where the authors emphasize that the band gap and exciton energy influence the PCE. Likewise, another ScYC­(OH)_2_ monolayer was investigated, exhibiting a low PCE of 23.44%.^93^ In addition, SiS_2_ and SiSe_2_ monolayers showed values of 3.02 and 21.25% at the BSE levels^95^ (see Table 1). Given these precedents, we obtained a value of 32.66% for NiO_2_, which makes this material comparable to other materials that were considered for photovoltaic applications.

It is important to notice that both PCE approximations has it is own limitations: (i) the SQ-limit assumes ideal carrier collection and perfect absorption (i.e all incident photons with energy equals or higher the optical band gap are absorbed); (ii) the modified SLME_max_ model presuming 100% photon absorption for excitations above the optical band gap, if light trapping schemes were applied,^97^ but also considers material-specific effects in the recombination fraction. Together, these yield upper-bound efficiencies up to 32.66% when a quasiparticle is included. In contrast, directly computed SLME efficiencies from actual absorption spectra and effective monolayer thickness are below 0.77%. This stark disparity underscores the limitations of single-layer absorbers. Therefore, 1T-NiO_2_ monolayer oxide material is better suited as a building block for stacked or heterostructured photovoltaic structures. In addition, in this low dimensional systems with high exciton binding energy $\left(E_{x_{b}}\right)$ , exciton dissociation can occur through the tunneling effect assisted by high electric fields.^98,99^

## 4 Conclusions

As shown in the previous sections, we provided a systematic characterization of 1T-NiO_2_ monolayer structural, optoelectronic, vibrational, and mechanical properties via first-principles calculations. All results are obtained by combining the DFT framework with a semiempirical MLWF-TB approach to describe the linear optical properties with excitonic effects, solving BSE. These results are used to estimate the solar harvesting efficiency using the PCE descriptor.

The monolayer’s dynamic stability is confirmed, as evidenced by the absence of imaginary frequencies in its phonon dispersion, and thermally stable at 300 K, as demonstrated by AIMD simulations. Furthermore, the material was shown to be mechanically stable and to exhibit an isotropic elastic response, with a Young’s modulus of 138.15 Nm^1–^.

Electronically, the material exhibits an indirect fundamental gap of 2.20 eV at the *G*_0_ *W*_0_ level. However, the most striking feature of the monolayer lies in its optical properties, which are dominated by strong electron–hole interactions. We identified an indirect excitonic ground state at 1.32 eV and a direct ground state at 1.34 eV, resulting in an exceptionally high exciton binding energy of 880 meV.

This high exciton binding energy, which far exceeds the thermal energy available at room temperature, indicates that exciton dissociation into free charge carriers is thermodynamically unfavorable. Although the excitonic optical gap (≈1.34 eV) is ideally positioned for solar absorption, leading to a theoretical photovoltaic efficiency limit (PCE_max_^SLME^) of 31.91%, this value must be interpreted with caution. It represents an idealized maximum that assumes efficient free-carrier generation, which is unlikely in this material.

Our findings indicate that the 1T-NiO_2_ monolayer is a stable 2D material. However, despite its strong visible-light absorption, it is an unsuitable candidate for conventional, free-carrier-based photovoltaic applications due to its strongly excitonic nature. However, its pronounced excitonic properties^100^ may make it promising for other applications in optoelectronics or photocatalysis,^101^ where the exciton itself (rather than its dissociated carriers) drives the desired process. Future studies may explore the use of heterostructures to facilitate exciton dissociation or directly harness these bound quasi-particle states.

From an experimental perspective, the synthesis of high-quality 1T-NiO_2_ monolayer could be pursued via CVD, exfoliation methods, or epitaxial growth on substrates that provide a close lattice match to minimize interfacial strain; hexagonal boron nitride (h-BN), quartz, or sapphire (Al_2_O_3_) are potential candidates for preserving the predicted electronic gap. To ensure high phase purity and thermal stability, growth parameters should be optimized to maintain oxygen stoichiometry and pressure-controlled, as the calculated high Young’s modulus (138.15 Nm^–^^1^) suggests the material can sustain significant epitaxial stress.

## Supplementary Material



## Notes

Crystal structure data in POSCAR format, complementary figures of the electronic band structure, and convergence tests (PDF)

## References

[1] Kalt G., Wiedenhofer D., Görg C., Haberl H. (2019). Conceptualizing energy
services: A review of energy and well-being along the Energy Service
Cascade. Energy Research & Social Science.

[2] Farghali M., Osman A. I., Mohamed I. M. A., Chen Z., Chen L., Ihara I., Yap P.-S., Rooney D. W. (2023). Strategies to save
energy in the context of the energy crisis: a review. Environmental Chemistry Letters.

[3] Novoselov K. S., Geim A. K., Morozov S. V., Jiang D., Zhang Y., Dubonos S. V., Grigorieva I. V., Firsov A. A. (2004). Electric Field Effect
in Atomically Thin Carbon Films. Science.

[4] Do
Nascimento Júnior C. A., Moujaes E. A., Piotrowski M. J., Caldeira Rêgo C. R., Guedes-Sobrinho D., Ribeiro Júnior L. A., da Silva Pereira T. A., Dias A. C. (2025). Unveiling the Stable Semiconducting 1T’-HfCl2
Monolayer: A New 2D Material. *ACS*. Omega.

[5] Dias A. C., Bragança H., de Mendonça J.
P. A., Da Silva J. L. F. (2021). Excitonic
Effects on Two-Dimensional Transition-Metal Dichalcogenide Monolayers:
Impact on Solar Cell Efficiency. ACS Applied
Energy Materials.

[6] Cavalheiro
Dias A., Almeida Cornélio C. D., Piotrowski M. J., Ribeiro Júnior L. A., de Oliveira Bastos C. M., Caldeira Rêgo C. R., Guedes-Sobrinho D. (2024). Can 2D Carbon
Allotropes Be Used as Photovoltaic Absorbers in Solar Harvesting Devices?. ACS Applied Energy Materials.

[7] Das S., Pandey D., Thomas J., Roy T. (2019). The Role of Graphene
and Other 2D Materials in Solar Photovoltaics. Adv. Mater..

[8] Butler K. T., Frost J. M., Skelton J. M., Svane K. L., Walsh A. (2016). Computational
materials design of crystalline solids. Chemical
Society Reviews.

[9] Zheng S., Shi X., Das P., Wu Z., Bao X. (2019). The Road Towards Planar
Microbatteries and Micro-Supercapacitors: From 2D to 3D Device Geometries. Adv. Mater..

[10] Chen X., Liu C., Mao S. (2020). Environmental Analysis with 2D Transition-Metal Dichalcogenide-Based
Field-Effect Transistors. Nano-Micro Lett..

[11] Wang S., Yan D., Ibarra Michel J., Corletto A., Wibowo A. A., Balendhran S., Lee H. Y., Byun S., Kim S., Crozier K. B., Sherrell P. C., Macdonald D., Bullock J. (2024). Improved Efficiency
in WSe2 Solar Cells Using Amorphous
InOx Heterocontacts. ACS Nano.

[12] Lin H., Buerki-Thurnherr T., Kaur J., Wick P., Pelin M., Tubaro A., Carniel F. C., Tretiach M., Flahaut E., Iglesias D., Vázquez E., Cellot G., Ballerini L., Castagnola V., Benfenati F., Armirotti A., Sallustrau A., Taran F., Keck M., Bussy C., Vranic S., Kostarelos K., Connolly M., Navas J. M., Mouchet F., Gauthier L., Baker J., Suarez-Merino B., Kanerva T., Prato M., Fadeel B., Bianco A. (2024). Environmental
and Health Impacts of Graphene and Other Two-Dimensional Materials:
A Graphene Flagship Perspective. ACS Nano.

[13] Ashby, M. F. Materials and the Environment: Eco-informed material choice, 2nd ed.; Butterworth-Heinemann: Oxford, 2013.

[14] Butler S. Z., Hollen S. M., Cao L., Cui Y., Gupta J. A., Gutiérrez H. R., Heinz T. F., Hong S. S., Huang J., Ismach A. F., Johnston-Halperin E., Kuno M., Plashnitsa V. V., Robinson R. D., Ruoff R. S., Salahuddin S., Shan J., Shi L., Spencer M. G., Terrones M., Windl W., Goldberger J. E. (2013). Progress, Challenges, and Opportunities
in Two-Dimensional Materials Beyond Graphene. ACS Nano.

[15] Manzeli S., Ovchinnikov D., Pasquier D., Yazyev O. V., Kis A. (2017). 2D transition
metal dichalcogenides. Nat. Rev. Mater..

[16] Bernardi M., Palummo M., Grossman J. C. (2013). Extraordinary
Sunlight Absorption
and One Nanometer Thick Photovoltaics Using Two-Dimensional Monolayer
Materials. Nano Letters.

[17] Gogotsi, Y. ; Anasori, B. The Rise of MXenes. In MXenes; Jenny Stanford Publishing, 2023; pp 3–11. DOI: 10.1201/9781003306511-2.

[18] Verger L., Natu V., Carey M., Barsoum M. W. (2019). MXenes: An Introduction
of Their Synthesis, Select Properties, and Applications. Trends in Chemistry.

[19] McGuire M. (2017). Crystal and
Magnetic Structures in Layered, Transition Metal Dihalides and Trihalides. Crystals.

[20] Kulish V. V., Huang W. (2017). Single-layer metal
halides MX2 (X = Cl, Br, I): stability and tunable
magnetism from first principles and Monte Carlo simulations. Journal of Materials Chemistry C.

[21] Lu A.-Y., Zhu H., Xiao J., Chuu C.-P., Han Y., Chiu M.-H., Cheng C.-C., Yang C.-W., Wei K.-H., Yang Y., Wang Y., Sokaras D., Nordlund D., Yang P., Muller D. A., Chou M.-Y., Zhang X., Li L.-J. (2017). Janus monolayers
of transition metal dichalcogenides. Nature
Nanotechnology.

[22] Aparicio-Huacarpuma B. D., Marinho E., Giozza W. F., Silva A. M. A., Kenfack-Sadem C., Dias A. C., Ribeiro L. A. (2025). 2D Janus SnSeS monolayers for solar
energy conversion: insights from DFT and excitonic analysis. Nanoscale.

[23] Bafekry A., Faraji M., Fadlallah M. M., Jappor H., Hieu N., Ghergherehchi M., Gogova D. (2022). Ab-initio-driven prediction of puckered
penta-like PdPSeX (X O, S, Te) Janus monolayers: Study on the electronic,
optical, mechanical and photocatalytic properties. Appl. Surf. Sci..

[24] Bafekry A., Faraji M., Fazeli S., Khan H. S., Fadlallah M. M., Stampfl C., Ghergherehchi M., Chang G. S., Shokri B. (2024). Controlling
the Electro-Optical Properties of an AlSb Monolayer with a DLHC Structure
through Phosphorus Alloying: A DFT Study. J.
Phys. Chem. C.

[25] Aparicio-Huacarpuma B.
D., Laranjeira J. A. d. S., Lopes Lima K. A., Moujaes E. A., Silva A. M. A., Ricardo Sambrano J., Cavalheiro Dias A., Ribeiro Júnior L. A. (2025). Optoelectronic and Excitonic Study
of XI 2 (X = Si, Ge, Sn, and Pb) Monolayers Envisaging Potential Technological
Applications. ACS Omega.

[26] Bafekry A., Naseri M., Faraji M., Fadlallah M. M., Hoat D. M., Jappor H. R., Ghergherehchi M., Gogova D., Afarideh H. (2022). Theoretical prediction of two-dimensional
BC2X (X = N, P, As) monolayers: ab initio investigations. Sci. Rep..

[27] Faraji M., Bafekry A., Fadlallah M. M., Jappor H., Nguyen C. V., Ghergherehchi M. (2022). Two-dimensional
XY monolayers (X = Al, Ga, In; Y =
N, P, As) with a double layer hexagonal structure: A first-principles
perspective. Appl. Surf. Sci..

[28] Bastos C. M. O., Besse R., Da Silva J. L. F., Sipahi G. M. (2019). Ab initio investigation
of structural stability and exfoliation energies in transition metal
dichalcogenides based on Ti-, V-, and Mo-group elements. Phys. Rev. Mater..

[29] Thomas N., Mathew S., Nair K., O’Dowd K., Forouzandeh P., Goswami A., McGranaghan G., Pillai S. (2021). 2D MoS2: structure, mechanisms, and photocatalytic
applications. Materials Today Sustainability.

[30] Radisavljevic B., Radenovic A., Brivio J., Giacometti V., Kis A. (2011). Single-layer MoS2 transistors. Nature Nanotechnology.

[31] Aparicio-Huacarpuma B. D., de Oliveira Bastos C. M., Laranjeira J. A. S., Mendonça F. L. L., Silva A. M. A., Sambrano R. J., Cavalheiro Dias A., Ribeiro Júnior L. A. (2025). Theoretical Prediction
of 2D Y2CTI (T = Br, Cl, F, H) Janus MXene Monolayers for Photovoltaic
Applications. ACS Omega.

[32] Zibouche N., Kuc A., Musfeldt J., Heine T. (2014). Transition-metal
dichalcogenides
for spintronic applications. Annalen der Physik.

[33] Pela R. R., Hsiao C.-L., Hultman L., Birch J., Gueorguiev G. K. (2024). Electronic
and optical properties of core–shell InAlN nanorods: a comparative
study via LDA, LDA-1/2, mBJ, HSE06, G0W0 and BSE methods. Phys. Chem. Chem. Phys..

[34] Oliveira M. J. T., Medeiros P. V. C., Sousa J. R. F., Nogueira F., Gueorguiev G. K. (2014). Optical and Magnetic Excitations of Metal-Encapsulating
Si Cages: A Systematic Study by Time-Dependent Density Functional
Theory. The Journal of Physical Chemistry C.

[35] Rao C. N. R. (1989). Transition
Metal Oxides. Annu. Rev. Phys. Chem..

[36] Santos W. O., Moucherek F. M. O., Dias A. C., Moreira E., Azevedo D. L. (2023). Structural,
optoelectronic, excitonic, vibrational, and thermodynamic properties
of 1T’-OsO_2_ monolayer via ab initio calculations. J. Appl. Phys..

[37] Santos W. O., Moucherek F. M. O., Dias A. C., Moreira E., Azevedo D. L. (2023). 1T’-RuO_2_ monolayer: First-principles study of excitonic, optoelectronic,
vibrational, and thermodynamic properties. J.
Mater. Res..

[38] Miró P., Audiffred M., Heine T. (2014). An atlas of two-dimensional materials. Chem. Soc. Rev..

[39] Hohenberg P., Kohn W. (1964). Inhomogeneous Electron Gas. Phys. Rev..

[40] Kohn W., Sham L. J. (1965). Self-Consistent
Equations Including Exchange and Correlation
Effects. Phys. Rev..

[41] Blöchl P. E. (1994). Projector Augmented-Wave
Method. PhysRevB.

[42] Kresse G., Joubert D. (1999). From ultrasoft pseudopotentials to
the projector augmented-wave
method. Physical Review B.

[43] Kresse G., Hafner J. (1993). Ab Initio Molecular
Dynamics for Open-Shell Transition
Metals. PhysRevB.

[44] Kresse G., Furthmüller J. (1996). Efficient
Iterative Schemes for Ab Initio Total-Energy
Calculations Using a Plane-Wave Basis Set. PhysRevB.

[45] Perdew J. P., Burke K., Ernzerhof M. (1996). Generalized
Gradient Approximation
Made Simple. Phys. Rev. Lett..

[46] Monkhorst H. J., Pack J. D. (1976). Special Points for
Brillouin-zone Integrations. Physical Review
B.

[47] Blum V., Gehrke R., Hanke F., Havu P., Havu V., Ren X., Reuter K., Scheffler M. (2009). *Ab-Initio* Molecular
Simulations with Numeric Atom-Centered Orbitals. computphyscommun.

[48] Havu V., Blum V., Havu P., Scheffler M. (2009). Efficient *O*(*N*) Integration for All-Electron Electronic
Structure Calculation Using Numeric Basis Functions. jcomputphys.

[49] Onida G., Reining L., Rubio A. (2002). Electronic excitations:
density-functional
versus many-body Green’s-function approaches. Rev. Mod. Phys..

[50] Hedin L. (1965). New Method
for Calculating the One-Particle Green’s Function with Application
to the Electron-Gas Problem. Phys. Rev..

[51] Shishkin M., Kresse G. (2006). Implementation and
performance of the frequency-dependent
GW method within the PAW framework. Physical
Review B.

[52] Perdew J. P., Zunger A. (1981). Self-Interaction Correction to Density-Functional Approximations
for Many-Electron Systems. Physical Review B.

[53] Bastos C. M. O., Sabino F. P., Sipahi G. M., Da Silva J. L. F. (2018). A Comprehensive
Study of *g* -Factors, Elastic, Structural and Electronic
Properties of III-V Semiconductors Using Hybrid-Density Functional
Theory. J. Appl. Phys..

[54] Mostofi A. A., Yates J. R., Lee Y.-S., Souza I., Vanderbilt D., Marzari N. (2008). wannier90: A tool for
obtaining maximally-localised
Wannier functions. Comput. Phys. Commun..

[55] Salpeter E. E., Bethe H. A. (1951). A Relativistic Equation
for Bound-State Problems. Phys. Rev..

[56] Dias A. C., Silveira J. F., Qu F. (2023). WanTiBEXOS:
A Wannier based Tight
Binding code for electronic band structure, excitonic and optoelectronic
properties of solids. Comput. Phys. Commun..

[57] ASTM International. Standard Tables for Reference Solar Spectral Irradiances: Direct Normal and Hemispherical on 37° Tilted Surface; ASTM G173-03(2020); ASTM International: West Conshohocken, PA, 2020 10.1520/G0173-03R20.

[58] Rozzi C. A., Varsano D., Marini A., Gross E. K. U., Rubio A. (2006). Exact Coulomb
cutoff technique for supercell calculations. Phys. Rev. B.

[59] Shockley W., Queisser H. J. (1961). Detailed Balance Limit of Efficiency ofp-nJunction
Solar Cells. J. Appl. Phys..

[60] Yu L., Zunger A. (2012). Identification of Potential
Photovoltaic Absorbers
Based on First-Principles Spectroscopic Screening of Materials. Phys. Rev. Lett..

[61] Wani A. F., Rani B., Sharopov U. B., Dhiman S., Kaur K. (2022). Thermoelectric
investigation of transition metal oxide NiO_2_: A first principles. International Journal of Energy Research.

[62] Rasmussen F. A., Thygesen K. S. (2015). Computational 2D
Materials Database: Electronic Structure
of Transition-Metal Dichalcogenides and Oxides. The Journal of Physical Chemistry C.

[63] Leong C. C., Pan H., Ho S. K. (2016). Two-dimensional
transition-metal oxide monolayers as
cathode materials for Li and Na ion batteries. Phys. Chem. Chem. Phys..

[64] Ouyang B., Lan G., Guo Y., Mi Z., Song J. (2015). Phase engineering of
monolayer transition-metal dichalcogenide through coupled electron
doping and lattice deformation. Appl. Phys.
Lett..

[65] Mouhat F., Coudert F.-X. (2014). Necessary and sufficient
elastic stability conditions
in various crystal systems. Phys. Rev. B.

[66] Born, M. ; Huang, K. Dynamical Theory of Crystal Lattices; Oxford University Press: New York, NY, 1996. DOI: 10.1093/oso/9780192670083.001.0001.

[67] Liu L., Wu J., Wu L., Ye M., Liu X., Wang Q., Hou S., Lu P., Sun L., Zheng J., Xing L., Gu L., Jiang X., Xie L., Jiao L. (2018). Phase-selective synthesis
of 1T’ MoS2 monolayers and heterophase bilayers. Nat. Mater..

[68] Han A., Zhou X., Wang X., Liu S., Xiong Q., Zhang Q., Gu L., Zhuang Z., Zhang W., Li F., Wang D., Li L.-J., Li Y. (2021). One-step synthesis
of single-site vanadium substitution in 1T-WS2 monolayers for enhanced
hydrogen evolution catalysis. Nat. Commun..

[69] Sun H., Liang Z., Shen K., Luo M., Hu J., Huang H., Zhu Z., Li Z., Jiang Z., Song F. (2018). Fabrication of NiSe2 by direct selenylation
of a nickel surface. Appl. Surf. Sci..

[70] Rajamathi M., Vishnu Kamath P., Seshadri R. (2000). Polymorphism in nickel hydroxide:
role of interstratification. Journal of Materials
Chemistry.

[71] Lee J., Park J. Y., Cho E. B., Kim T. Y., Han S. A., Kim T., Liu Y., Kim S. K., Roh C. J., Yoon H., Ryu H., Seung W., Lee J. S., Lee J., Kim S. (2017). Reliable Piezoelectricity
in Bilayer WSe2 for Piezoelectric Nanogenerators. Adv. Mater..

[72] Michels L., Cygan B., Pawlyta M., Jezierski J., Götz A., Akola J. (2024). Graphite nucleation on (Al, Si, Mg)-nitrides:
Elucidating the chemical interactions and turbostratic structures
in spheroidal graphite cast irons. Carbon.

[73] Li T. (2012). Ideal strength
and phonon instability in single-layer MoS2. Phys. Rev. B.

[74] Sheng X.-F., Rao X.-X., Ke C., Kang W.-B. (2022). 2D plane XP2 (X
= Ni, Pd, Pt) with narrow band gaps, ultrahigh carrier mobility and
high electrical transport performance. Appl.
Surf. Sci..

[75] Fang W., Xiao X., Wei H., Chen Y., Li M., He Y. (2022). The elastic, electron, phonon, and vibrational properties of monolayer
XO2 (X = Cr, Mo, W) from first principles calculations. Materials Today Communications.

[76] Qu L.-H., Yu J., Mu Y.-L., Fu X.-L., Zhong C.-G., Min Y., Zhou P.-X., Zhang J.-M., Zou Y.-Q., Lu T.-S. (2019). Strain
tunable structural, mechanical and electronic properties of monolayer
tin dioxides and dichalcogenides SnX2 (X O, S, Se, Te). Mater. Res. Bull..

[77] Priyanka, Ritu, Kumar V., Kumar R., Chand F. (2024). First principle
calculations to explore the electronic, mechanical
and optical properties of 2D NiX2 (X = O, S, Se) monolayers. Physica B: Condensed Matter.

[78] Martins N. F., Laranjeira J. A. S., Aparicio-Huacarpuma B.
D., Ribeiro Junior L. A., Sambrano J. R. (2025). Computational Characterization of the Novel Ta2Se2C
Transition Metal Carbo-Chalcogenide as Anode Material for Li and Na-Ion
Batteries. The Journal of Physical Chemistry
C.

[79] Menéndez-Proupin E., Morell E. S., Marques G. E., Trallero-Giner C. (2024). Lattice vibration
modes and electron–phonon interactions in monolayer vs. bilayer
of transition metal dichalcogenides. *RSC*. Advances.

[80] Das A., Singh D., Saini C. P., Ahuja R., Kaur A., Aliukov S. (2020). Orbital hybridization-induced band offset phenomena
in Ni_x_Cd_1‑x_O thin films. Nanoscale.

[81] Zaanen J., Sawatzky G. A., Allen J. W. (1985). Band gaps and electronic structure
of transition-metal compounds. Phys. Rev. Lett..

[82] Riis-Jensen A. C., Deilmann T., Olsen T., Thygesen K. S. (2019). Classifying the
Electronic and Optical Properties of Janus Monolayers. ACS Nano.

[83] Aparicio-Huacarpuma B. D., Pereira Júnior M. L., Silva A. M. A., Dias A. C., Ribeiro Júnior L. A. (2025). Solar Harvesting Efficiency of Janus
M2CTT’(M = Y, Sc; T/T’ = Br, Cl, F) MXene Monolayers
for Photovoltaic Applications. ACS Applied Energy
Materials.

[84] Aparicio-Huacarpuma B. D., Pereira M. L., Piotrowski M. J., Rêgo C. R. C., Guedes-Sobrinho D., Ribeiro L. A., Dias A. C. (2025). Enhanced
solar harvesting efficiency in nanostructured MXene monolayers based
on scandium and yttrium. Nanoscale.

[85] Park S., Mutz N., Schultz T., Blumstengel S., Han A., Aljarb A., Li L.-J., List-Kratochvil E. J. W., Amsalem P., Koch N. (2018). Direct determination
of monolayer
MoS2and WSe2exciton binding energies on insulating and metallic substrates. 2D Materials.

[86] Aparicio-Huacarpuma B. D., Villegas C. E. P., Meira G. M. C., Bastos C. M. O., Seridonio A. C. F., Dias A. C., Ribeiro L. A., Marinho E. (2026). Structural effects
on mechanical and optoelectronic properties of S-doped graphyne. Phys. Rev. B.

[87] Kumar N., Kolos M., Bhattacharya S., Karlický F. (2024). Excitons,
optical spectra, and electronic properties of semiconducting Hf-based
MXenes. J. Chem. Phys..

[88] Liu H.-L., Shen C.-C., Su S.-H., Hsu C.-L., Li M.-Y., Li L.-J. (2014). Optical properties
of monolayer transition metal dichalcogenides
probed by spectroscopic ellipsometry. Appl.
Phys. Lett..

[89] Saleem M., Atiq S., Ramay S. M., Mahmood A., ur Rehman A., Saad Khaliq H., Siddiqi S. A. (2020). Investigations on electronic and
optical properties of Ag:MoS2 co-sputtered thin films. Chem. Phys. Lett..

[90] Akhtar P., Khan M. J. I., Kanwal Z., Ramay S. M., Mahmood A., Saleem M. (2021). Ab-initio and experimental investigations
on Au incorporated
MoS2 for electronic and optical response. J.
Alloys Compd..

[91] Rühle S. (2016). Tabulated
values of the Shockley–Queisser limit for single junction solar
cells. Solar Energy.

[92] Aparicio-Huacarpuma B.
D., Laranjeira J. A., Martins N. F., Gonzalo F. M., Lima K. A., Silva A. M., Sambrano J. R., Junior L. A. R. (2025). Photovoltaic
and gas sensing properties of the novel bimetallic Janus ScNbCO2 MXene. Surfaces and Interfaces.

[93] Aparicio-Huacarpuma B. D., Marinho E., Laranjeira J. A., Giozza W. F., Silva A. M., Dias C. A., Ribeiro L. A. (2025). Two-dimensional
functionalized mm’ct2 (m= sc;
m’= y; t= br, cl, f, h, i, o, oh, s, se, te) mxene monolayers
for photovoltaic applications. J. Phys. Chem.
C.

[94] Moujaes E. A., Dias A. C. (2023). On the excitonic
effects of the 1T and 1OT phases of
PdS2, PdSe2, and PdSSe monolayers. J. Phys.
Chem. Solids.

[95] Shuai L., Fujun L., Li J., Chaldyshev V. V., Dias A. C. (2025). Photocatalytic water splitting and excitonic effects
of novel SiS2/SiSe2 heterojunction. Appl. Surf.
Sci..

[96] Huacarpuma B. D. A., Irfan M., Huayhua C. A. V., de Mendonça F. L. L., Bastos C. M., Dias A. C., Ribeiro L. A. (2026). Electronic structure
and light-harvesting efficiency of Janus XSO (X = Sn, Ge) monolayers. Physica E: Low-dimensional Systems and Nanostructures.

[97] Jariwala D., Davoyan A. R., Wong J., Atwater H. A. (2017). Waals Materials
for Atomically-Thin Photovoltaics: Promise and Outlook. ACS Photonics.

[98] Massicotte M., Vialla F., Schmidt P., Lundeberg M. B., Latini S., Haastrup S., Danovich M., Davydovskaya D., Watanabe K., Taniguchi T., Fal’ko V. I., Thygesen K. S., Pedersen T. G., Koppens F. H. L. (2018). Dissociation
of two-dimensional excitons in monolayer WSe2. Nat. Commun..

[99] Mahmood A., Gao M., Greenberg M., Pivrikas A., Gentle I., Philippa B. (2025). Charge transport
and electric field-dependent exciton dissociation in organic semiconductors
measured using accumulation at an insulator–semiconductor layer. Appl. Phys. Lett..

[100] Wang G., Chernikov A., Glazov M. M., Heinz T. F., Marie X., Amand T., Urbaszek B. (2018). Colloquium: Excitons
in atomically thin transition metal dichalcogenides. Rev. Mod. Phys..

[101] Wang H., Jin S., Zhang X., Xie Y. (2020). Excitonic
Effects in Polymeric Photocatalysts. Angewandte
Chemie International Edition.

