# Supplementary material for: Excitonic, Optical, and Photovoltaic Properties of the 1T-NiO2 Monolayer
Source: ACS Omega. 2026 Mar 27;11(13):20751–62. doi: 10.1021/acsomega.5c12803 (PMC13062993; doi:10.1021/acsomega.5c12803)
Supplement: Supplementary file 1 [file ao5c12803_si_001.pdf]

Supporting Information: Excitonic, Optical and Photovoltaic Properties of the 1T-NiO_2 Monolayer

Israel da Silva Oliveira,[*,†,‡] Bill D. Aparicio Huacarpuma,[†,‡] Carlos M. de Oliveira Bastos,[¶] Mariana Lumi Ichihara Sado,[§] Alysson M. Almeida Silva,[§] Luiz Antônio Ribeiro Júnior,[‖,⊥] and Alexandre Cavalheiro Dias[*,#,⊥]

†*Institute of Physics, University of Brasília, Brasília 70919-970 DF, Brazil*

‡*Computational Materials Laboratory, LCCMat, Institute of Physics, University of Brasília, 70910-900 Brasília, Brazil*

¶*Institute of Physics and International Center of Physics, University of Brasília, Brasília 70919-970 DF, Brazil*

§*University of Brasília, College of Technology, Department of Mechanical Engineering, 70910-900, Brasília, Federal District, Brazil.*

‖*University of Brasília, Institute of Physics, 70910-900, Brasília, Federal District, Brazil*

⊥*Computational Materials Laboratory, LCCMat, Institute of Physics, University of Brasília, 70910-900, Brasília, Federal District, Brazil*

#*University of Brasília, Institute of Physics and International Center of Physics, Brasília 70919-970, Federal District, Brazil*

E-mail: israeldaoliveira@gmail.com; alexandre.dias@unb.br

Contents

S1 POSCAR of Optimized Geometries **S3**

S2 Electronic Band Structure **S4**

S3 G_0W_0 Convergence **S5**

S4 Vacuum Convergence **S6**

S1 POSCAR of Optimized Geometries

The optimized geometry coordinates for the 1T-NiO_2 monolayer are presented below in VASP POSCAR format.

```
Ni1 O2
   1.0000000000000000
     2.8167348379438053    0.0000000000000000    0.0000000000000000
    -1.4083674189223054    2.4393639253797508    0.0000000000000000
     0.0000000000000000    0.0000000000000000   16.8988208771000004
   Ni    O
     1     2
Direct
     0.0000000000000000    0.0000000000000000    0.5000000000000000
     0.6666666870000029    0.3333333429999996    0.5558011059147674
     0.3333333429999996    0.6666666870000029    0.4441989230852315
```

Table S1: Optimized structural parameters and cohesive energy for the 1T-NiO_2 monolayer.

**Phase**	**a (Å)**	**b (Å)**	$\boldsymbol{\alpha}$ (°)	$\boldsymbol{\beta}$ (°)	$\boldsymbol{\gamma}$ (°)	t (Å)	$\mathbf{E}_{\mathrm{coh/atom}}$ (eV)
1T	2.817	2.817	90	90	120	1.886	-4.376

S2 Electronic Band Structure

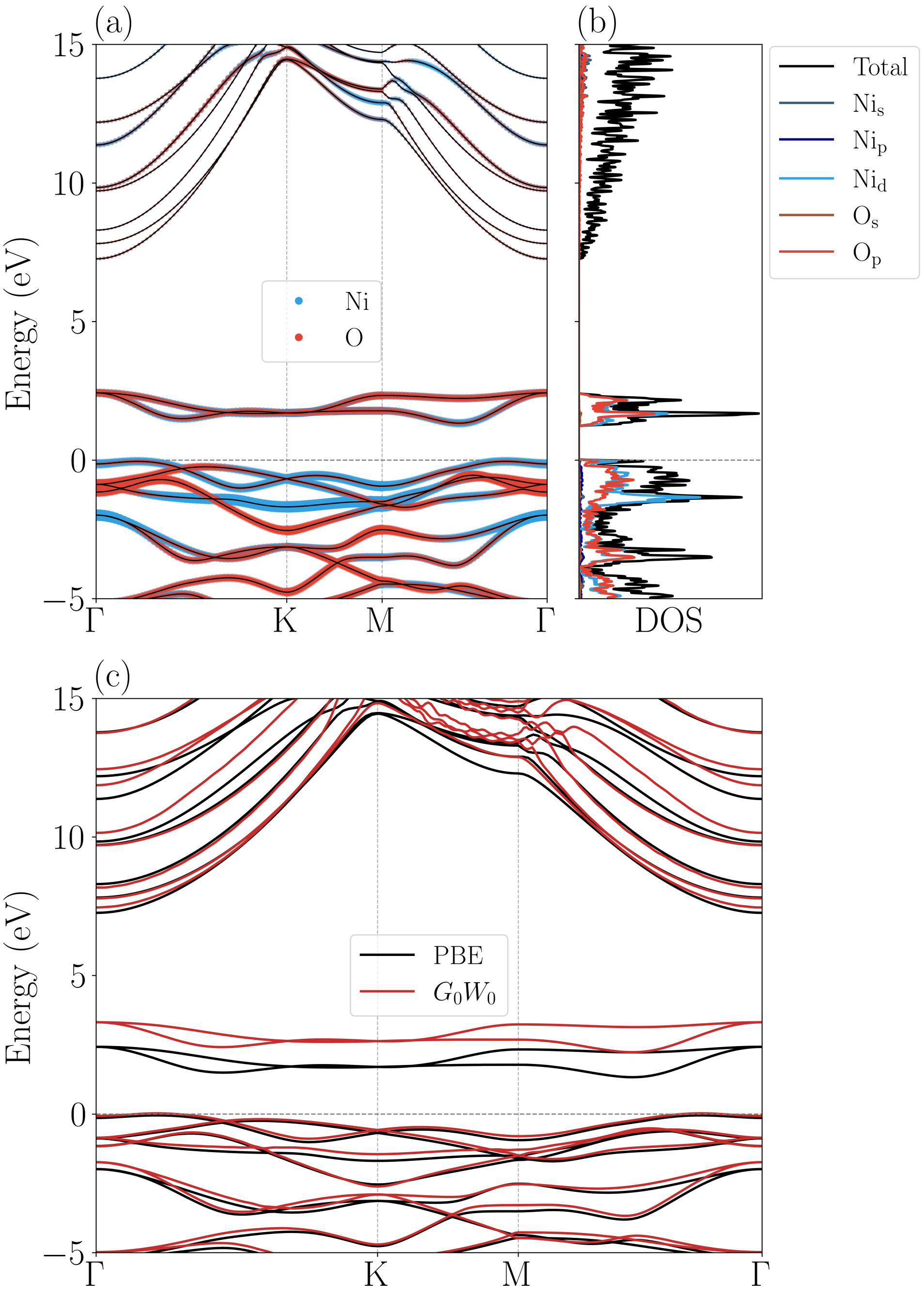

Figure S1: (a) Projected electronic band structure showing the contributions of Ni and O atoms, calculated within the PBE functional. (b) Total and projected density of states (DOS), displaying the dominant orbital contributions of Ni and O at the PBE level. (c) Comparison of PBE (black curves) and G_0W_0 (red curves) electronic band structures.

S3 G_0W_0 Convergence

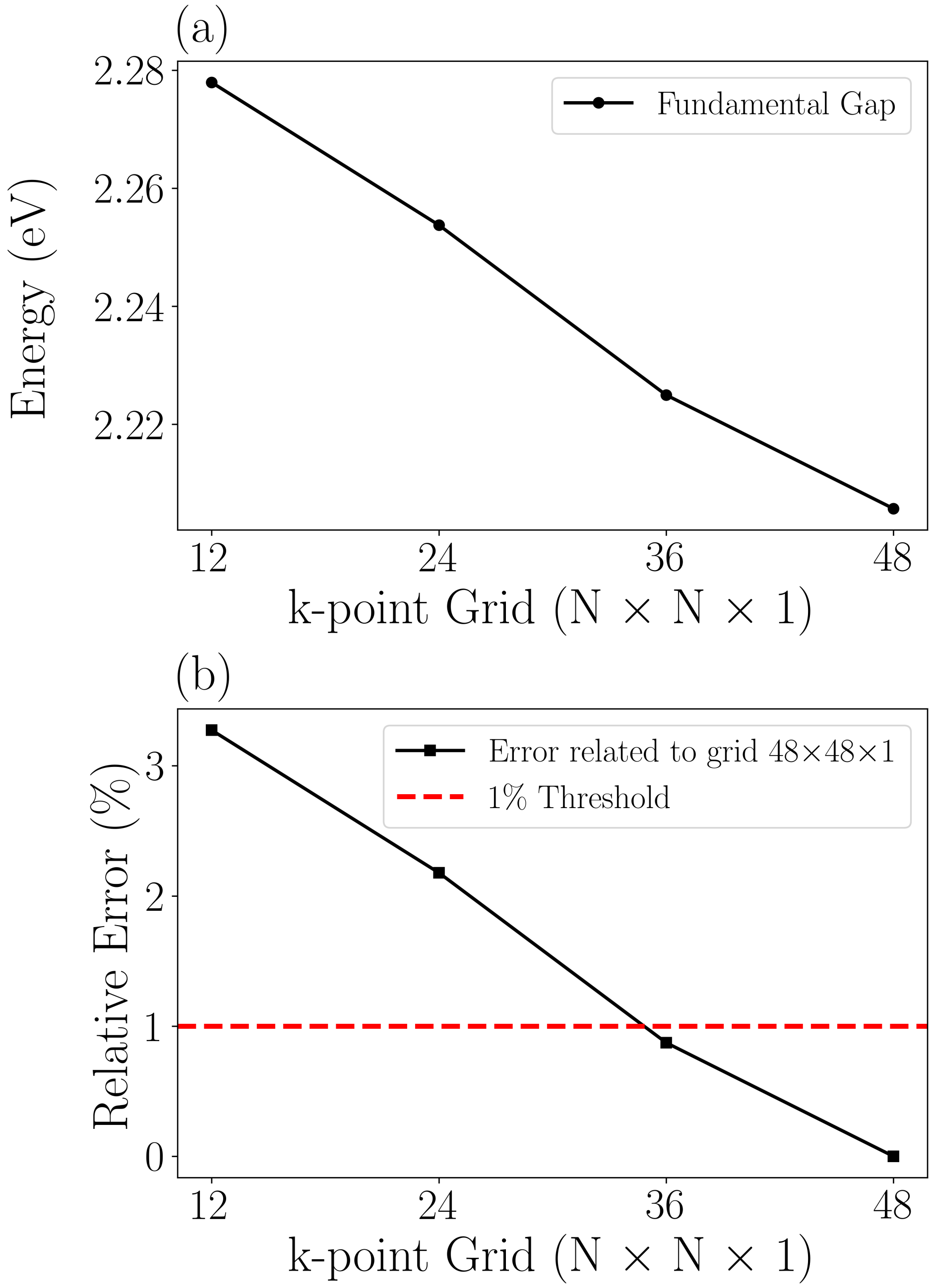

Figure S2: Convergence analysis of the G_0W_0 quasiparticle band gap with respect to the **k**-point sampling density. (a) Evolution of the fundamental gap energy as a function of the grid size ($N \times N \times 1$). (b) Relative percentage error calculated with respect to the finest grid considered ($48 \times 48 \times 1$). The red dashed line indicates a convergence threshold of 1%, which is achieved with the $36 \times 36 \times 1$ grid.

S4 Vacuum Convergence

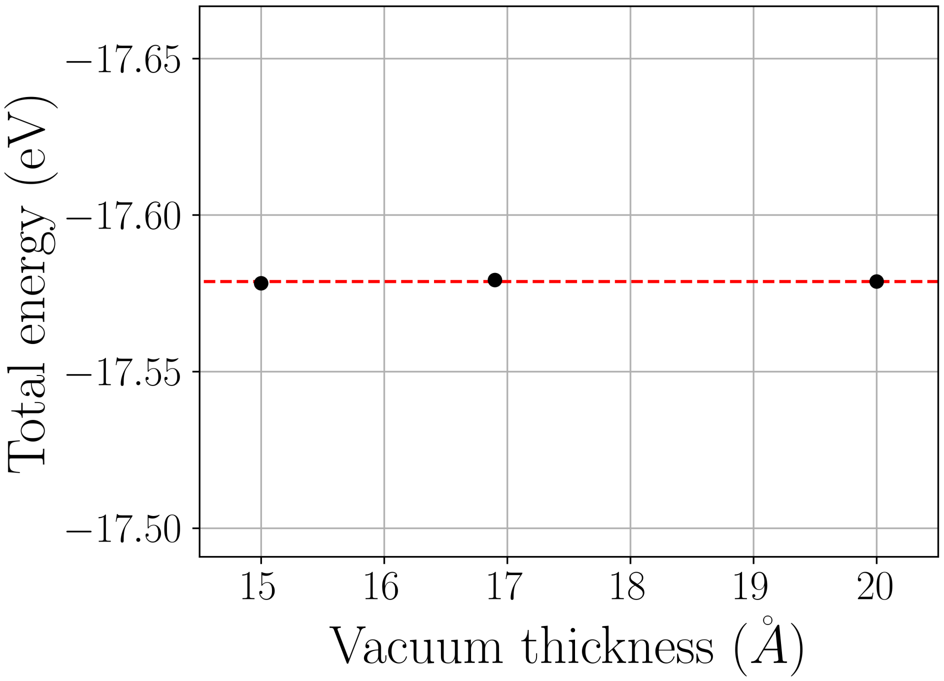

Figure S3: Total energy convergence as a function of vacuum distance thickness.
